# Characteristics and predictive factors of severe or fatal suicide outcome in patients hospitalized due to deliberate self-poisoning

**DOI:** 10.1371/journal.pone.0276000

**Published:** 2022-11-03

**Authors:** Stefanie Geith, Maja Lumpe, Johannes Schurr, Christian Rabe, Armin Ott, Tobias Zellner, Michael Rentrop, Florian Eyer

**Affiliations:** 1 Division of Clinical Toxicology and Poison Control Centre Munich, Department of Internal Medicine II, School of Medicine, Technical University of Munich, Munich, Germany; 2 Lungenfachkliniken München-Gauting, Gauting, Germany; 3 Staburo GmbH, Munich, Germany; 4 Clinic and Polyclinic for Psychiatry and Psychotherapy, Klinikum rechts der Isar, Technical University of Munich, Munich, Germany; 5 kbo-Inn-Salzach Clinic, Wasserburg am Inn, Germany; Mahidol University, Faculty of Tropical Medicine, THAILAND

## Abstract

Prediction of clinical course of intoxication is essential for timely initiation of appropriate medical treatment in patients hospitalized due to suicidal self-poisoning. In this retrospective single-centre study in patients hospitalized due to suicidal poisoning in a specialized clinical toxicology unit, we aimed to identify predictive factors associated with severe or fatal course of self-poisoning. All patients underwent at least one psychiatric exploration during their inpatient stay. Severity of poisoning was assessed on admission and after 24 hours according to the Poison Severity Score index (PSS). Spearman’s rank correlation coefficient was used to test the association of PSS with sociodemographic, anamnestic and (pre-)clinical parameters. Multivariable binomial logistic regression analysis was performed to determine predictive factors for severe and/or fatal self-poisoning. 1090 patients were included in the study. Median age was 39 years (range 13–91), 66.7% of patients were female. PSS was classified in the majority as “minor” (n = 558, 51.2%) or “moderate” (n = 264, 24.2%). 61 patients (5.6%) had PSS “severe”; 14 patients (1.3%) died. A higher severity of poisoning positively correlated with duration of inpatient therapy (p<0.001, Spearman’s rho = 0.454) and duration of ventilation (p<0.001, rho = 0.474), and it inversely correlated with initial Glasgow Coma Scale (GCS) score (p<0.001, rho = -0.437). Multivariable analysis identified no alcohol co-ingestion (OR 3.23; 95%CI 1.3, 8.07; p = 0.012) and self-poisoning with non-medicinal substances (OR 5.4; 95%CI 1.78, 16.34; p = 0.003) as factors predictive for “severe” or “fatal” suicide outcome. In contrast, female gender (OR 0.4; 95%CI 0.2, 0.81; p = 0.011), not using an antidepressant as the method for self-poisoning (OR 0.27; 95%CI 0.12, 0.59; p = 0.001) and a higher initial GCS score (OR 0.79; 95%CI 0.73, 0.85; p<0.001) reduced the risk of a severe or fatal course of self-poisoning. The conclusion for clinical practice is that male patients hospitalized due to self-poisoning, with a low initial GCS score, who did not co-ingest alcohol, attempted suicide with non-pharmaceutical substances or antidepressants are at a higher risk of severe/fatal outcome of suicide. Determination of these risk factors at admission could be potentially used to guide treatment intensification in patients hospitalized due to deliberate self-poisoning.

## Introduction

Deliberate self-poisoning is a common reason for emergency department (ED) and intensive care unit (ICU) admissions accounting for 0.4% to 10% of all ED attendances [1–9] and 2.3% to 17.3% of all ICU admissions [9–12]. Although the in-hospital mortality rate is rather low at 0.5% to 4% reported in the literature [9, 10, 12, 13], it is crucial to identify patients at risk of death. However, it is often difficult, even for an experienced emergency or intensive care physician, to predict the clinical course of an intoxication at admission. Especially in the early phase of intoxication, where the clinical severity may not have reached its peak, a valid risk assessment may be challenging at the time of admission. Therefore, identification of factors associated with the severity of poisoning would be helpful to timely initiate medically indicated steps, such as an intensive care transfer, protective intubation or establishing the indication for antidote administration. Thus, this study aims to determine the characteristics and potential predictive factors for a severe or fatal course of poisoning.

## Materials and methods

### Study design

For this retrospective single-centre study, electronic records of patients treated between 1 January 2012 and 31 December 2016 in the Department of Clinical Toxicology within a tertiary university hospital in Bavaria, Germany, were queried to identify the cases meeting the following criteria: (i) self-poisoning based on suicide-related behavior (SRB) and (ii) exploration by a board-certified psychiatrist at least once during the inpatient stay in non-fatal cases. SRB was defined according to the definition of Silverman et al. [14, 15] which included the terms self-harm, suicide attempt and suicide. (Para-)suicidal intent was confirmed within the psychiatric consultation or assumed in the case of fatal outcome, when suicide intention seemed most probable (e.g., when a suicide note was left, or relatives confirmed patient’s wish to die). Due to the retrospective character of the study, a written informed consent was not obtained from patients. The study was approved by the Ethic Committee of the University Hospital (No. 270/16s), and it was conducted in compliance with the Declaration of Helsinki.

### Data analysis

Poison Severity Score index (PSS, [16]) was used to determine the severity of poisoning based on the data in the patients’ medical records. PSS was assessed on admission and after 24 hours; only the most serious PSS score during the inpatient stay was used for the analysis. The remaining data, including 60 variables, were directly transferred from patients’ medical records into a Microsoft Access database. In case of more than one clinical admission due to SRB in the same individual, we only included the chronologically first case. For age group analysis, the following four age groups were defined: <18 years, 18–44 years, 45–64 years, and >64 years. Patients were stratified into four groups based on the rescue time until hospital admission (<1h, 1-3h, 3-6h and >6h). These categories were set arbitrarily to investigate whether a longer rescue time correlated with a worse outcome and to differentiate between suicidal and a parasuicidal acts. We presumed that patients committing parasuicidal acts were more likely to call for help or rescue on their own and thus have a shorter rescue time. In contrast, patients with a true intention to die could be found later because of the severity of the poisoning or because they arranged their suicide in a way that they were difficult to find.

Both surviving and fatal suicide attempts were included in the analysis. Cases were considered fatal regardless of whether death occurred due to a direct toxic effect or to a further complication such as aspiration pneumonia or sepsis (information on the actual cause of death was not collected from the autopsy report). Pre-existing or new psychiatric disorders (PD), as diagnosed by psychiatrist before and after hospitalization, respectively, were categorized in accordance with the ICD classification of mental and behavioral disorders; up to three pre-existing PDs were transferred from the psychiatric consultation reports (S1 Table). To investigate the relationship between a specific PD and the severity of an intoxication, only one PD was assigned to each patient, regardless of whether a PD was previously known or newly diagnosed during the inpatient stay: (i) if the PDs diagnosed before and after hospitalization were the same, then this PD was used; (ii) if the PD diagnosed before and after hospitalization were different, then term “combined PD” was used. Since our centre uses standardized psychiatric consultation reports, patients with no information on PD were treated as not diagnosed with PD for the purpose of the present analysis.

Multiple of maximum daily dose (MDD) was used to describe the amount of the substance used in self-poisoning, and it indicates how many times the maximum daily dose was crossed. In cases where two or more substances were used, only the higher multiple of MDD was used in the analysis, e.g., if a patient took 4800 mg ibuprofen (multiple of MDD of 2, given the recommended MDD of 2400 mg), and 400 mg citalopram (multiple of MDD of 10, given the recommended MDD of 40 mg), then multiple of MDD for citalopram (10) was used.

### Statistical analysis

Data was analyzed descriptively using SPSS Statistics for Windows (version 25, IBM Corp., Armonk, N.Y., USA) and R (version 3.5.2; R Foundation for Statistical Computing, Vienna, Austria). Analyses of the predominantly nominal scaled data were performed with nonparametric tests and were exploratory in nature. Quantitative data was described as mean ± standard deviation and categorical variables are presented as absolute and relative frequencies. We analyzed nominal and ordinal variables by using Pearson’s Chi-squared test or Fisher’s exact test for sample sizes of five or fewer and interval scaled variables by Student’s t-test. We considered p-values ≤0.05 statistically significant. In the case of multiple testing, we did not adjust p-values given the descriptive nature of evaluation.

Multivariable binomial logistic regression analysis was used to determine predictive factors for severe and/or fatal self-poisoning. For the purpose of regression analysis, cases with PSS scores "severe" and "fatal" were combined due to the small number of cases. Variables considered as potential predictors for severe or fatal outcome of poisoning were selected based on their medical relevance, availability in everyday clinical practice and exploratory analyses regarding the intoxication severity performed in this study. A stepwise selection procedure using the Akaike Information Criterion (AIC) was performed to identify the preferred multivariable model by comparing the informative efficacy and goodness of fit. Selected variables were examined according to their effect size in terms of their predictive value for severe and/or fatal self-poisoning. A regression model emerged that mapped predictive factors for or against a severe and/or fatal course, depending on their odds ratios (OR).

## Results

### Patient characteristics

**Fig 1:**
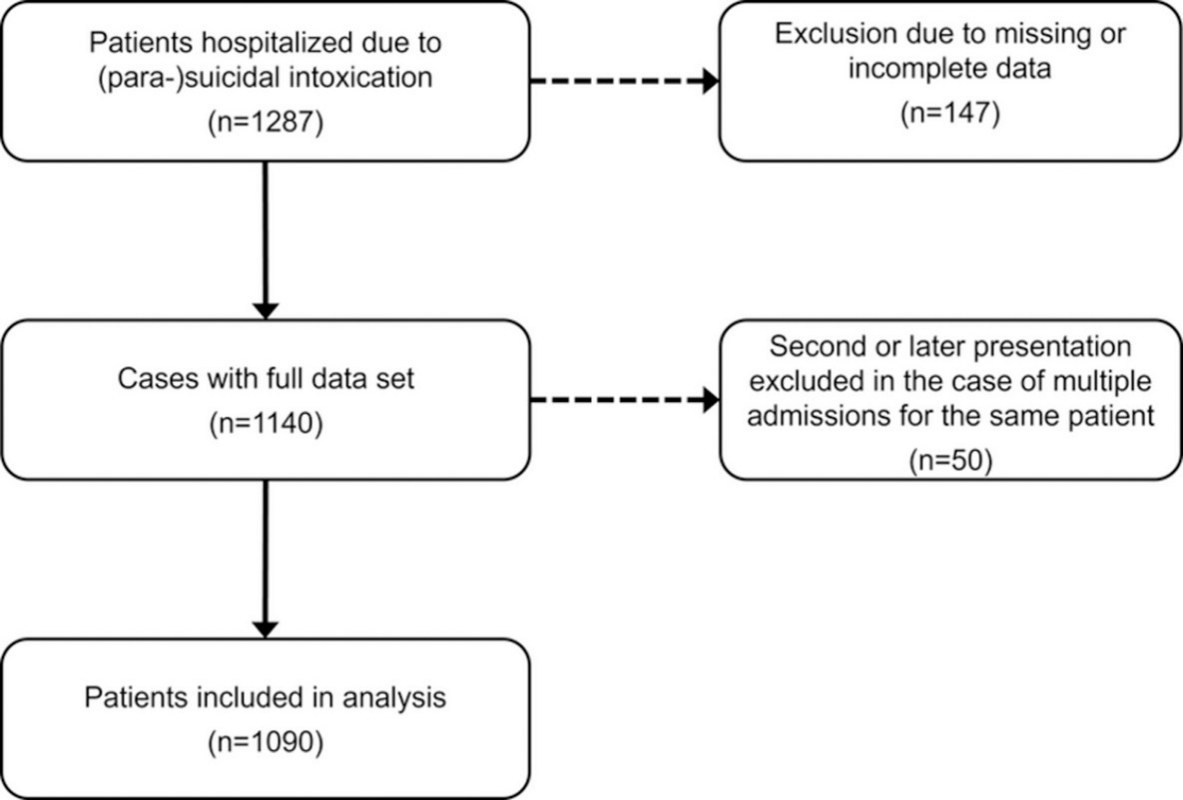
CONSORT diagram.

Out of 29 434 patients treated in our Department of Clinical Toxicology in the period from 1 January 2012 to 31 December 2016, 1287 were admitted due to suicidal intoxication. After excluding 147 cases with incomplete data and considering only the chronologically first presentation in case of multiple admission, 1090 patients were included in the final analysis (Fig 1). According to PSS, minor poisonings predominated (n = 558/1090, 51.2%) over moderate (n = 264, 24.2%) or asymptomatic (PSS: none) intoxications (n = 193, 17.7%), whereas severe and fatal poisonings were rather rare (n = 61, 5.6%; and n = 14, 1.3%, respectively).

### Relationship between socio-demographic parameters and severity of poisoning

Table 1 shows socio-demographic parameters grouped by the severity of the poisoning (the results of pairwise comparisons are shown in S4 Table). There were significant differences between the degrees of severity in terms of gender, age groups, average age, and parenthood. In particular, the male gender dominated in case of lethal poisonings whereas in the PSS: none, minor, and moderate cases, women predominated. Only in cases of severe poisoning both sexes were almost equally represented. We detected a weak positive correlation between age group and the severity of poisoning (Spearman rho = 0.198). When looking at the gender-dependent age distribution, it is noticeable that men (except for severe cases) were most often older than women with a trend that the average age increased with poison severity (S1 Fig). Patients with children were overrepresented in PSS groups moderate and severe. There were no significant differences between severity of poisoning groups regarding the employment status.

**Table 1:** Relationship between sociodemographic parameters and severity of poisoning.

	None (n = 193)	Minor (n = 558)	Moderate (n = 264)	Severe (n = 61)	Fatal (n = 14)	Total (n = 1090)	p-value
**Sex**							
Male	63 (32.6)	171 (30.6)	91 (34.5)	30 (49.2)	8 (57.1)	363 (33.3)	0.016
Female	130 (67.4)	387 (69.4)	173 (65.5)	31 (50.8)	6 (42.9)	727 (66.7)	
**Age**							
Mean	34.7	39.6	44.8	44.3	56.4	40.5	<0.001
Median (min, max)	32 (13, 90)	38 (13, 85)	43.5 (13, 91)	45 (15, 75)	53 (15, 90)	39 (13, 91)	
**Age group**							
<18	19 (9.8)	27 (4.8)	9 (3.4)	2 (3.3)	1 (7.1)	58 (5.3)	<0.001
18–44	125 (64.8)	321 (57.5)	129 (48.9)	27 (44.3)	1 (7.1)	603 (55.3)	
45–64	40 (20.7)	163 (29.2)	84 (31.8)	24 (39.3)	7 (50)	318 (29.2)	
>64	9 (4.7)	47 (8.4)	42 (15.9)	8 (13.1)	5 (35.7)	111 (10.2)	
**Employed**	60 (39.2)	203 (43.8)	82 (35.8)	20 (37.0)	3 (30.0)	368 (40.4)	0.299
Missing	40	94	35	7	4	180	
**Children**	63 (36.2)	227 (43.8)	123 (52.1)	34 (58.6)	4 (50.0)	451 (45.4)	0.004
Missing	19	40	28	3	6	96	

Unadjusted p-values are shown.

### Relationship between anamnestic parameters and severity of poisoning

Table 2 displays the list of anamnestic parameters by the severity of poisoning (the results of pairwise comparisons are shown in S4 Table). Significant differences were found with respect to cardiovascular, neurological, neoplastic, and metabolic preexisting conditions, the proportion of assigned different psychiatric diagnoses (data on the concomitant medication has not been collected in the study), the presence of suicidal ideation or a suicide note, and the different trigger factors at each severity level. Among the assigned PD (consolidated preexisting and newly diagnosed PD), stress disorders dominated among the asymptomatic cases (PSS: none), while mood disorders were observed more frequently in more severe cases. Furthermore, the proportion of patients with suicidal thoughts prior to current suicide attempt or who left a suicide note predominated in each case within the severe cases. The average number of prior suicide attempts was lower in the fatal case, although not statistically significantly different. However, the severity of poisoning did not correlate with the number of preexisting PD and number of SRB (Spearman’s rho 0.016 and 0.032, respectively). As far as trigger factors are concerned, in the milder severity levels (PSS none, minor) mostly partner conflicts were named, while within the higher severity levels (PSS moderate, severe, fatal) "no trigger factor" as well as family and health problems were noted.

**Table 2:** Relationship between patient related information and severity of poisoning.

	None (n = 193)	Minor (n = 558)	Moderate (n = 264)	Severe (n = 61)	Fatal (n = 14)	Total (n = 1090)	p-value
**Concomitant disease**							
Cardiovascular	20 (10.4)	58 (10.4)	56 (21.2)	11 (18.0)	3 (21.4)	148 (13.6)	<0.001
Pulmonary	9 (4.7)	32 (5.7)	16 (6.1)	1 (1.6)	2 (14.3)	60 (5.5)	0.329
Neurological	19 (9.8)	66 (11.8)	51 (19.3)	8 (13.1)	3 (21.4)	147 (13.5)	0.017
Neoplastic	3 (1.6)	22 (3.9)	13 (4.9)	2 (3.3)	3 (21.4)	43 (3.9)	0.020
Infectious	2 (1.0)	16 (2.9)	4 (1.5)	1 (1.6)	0 (0)	23 (2.1)	0.572
Endocrine	14 (7.3)	71 (12.7)	51 (19.3)	11 (18.0)	2 (14.3)	149 (13.7)	0.003
**Preexisting PD known**	112 (58.0)	376 (67.4)	166 (62.9)	43 (70.5)	8 (57.1)	705 (64.7)	0.125
**Number of preexisting PD**							
0	81 (42.0)	182 (32.6)	98 (37.1)	18 (29.5)	6 (42.9)	385 (35.3)	0.609
1	71 (36.8)	251 (45.0)	113 (42.8)	27 (44.3)	8 (57.1)	470 (43.1)	
2	29 (15.0)	86 (15.4)	33 (12.5)	11 (18.0)	0 (0)	159 (14.6)	
3	12 (6.2)	39 (7.0)	20 (7.6)	5 (8.2)	0 (0)	76 (7.0)	
**Assigned PD (consolidated preexisting and newly diagnosed PD)**							
Addiction	3 (1.6)	22 (3.9)	3 (1.1)	1 (1.6)	2 (14.3)	31 (2.8)	<0.001
Schizophrenia	7 (3.6)	22 (3.9)	19 (7.2)	4 (6.6)	0 (0)	52 (4.8)	
Mood disorder	43 (22.3)	148 (26.5)	104 (39.4)	24 (39.3)	5 (35.7)	324 (29.7)	
Stress disorder	45 (23.3)	101 (18.1)	32 (12.1)	7 (11.5)	0 (0)	185 (17.0)	
Personality disorder	17 (8.8)	33 (5.9)	14 (5.3)	2 (3.3)	1 (7.1)	67 (6.1)	
Other PD	1 (0.5)	5 (1.0)	2 (0.8)	0 (0)	1 (7.1)	9 (0.8)	
Combined PD	62 (32.1)	199 (35.7)	75 (28.4)	21 (34.4)	0 (0)	357 (32.8)	
No PD	15 (7.8)	28 (5.0)	15 (5.7)	2 (3.3)	5 (35.7)	65 (6.0)	
**Number of SRB**							
Mean	1.4	1.5	1.8	1.8	1.0	1.5	0.296
Median (min. max)	1 (1, 6)	1 (1, 10)	1 (1, 86)	1 (1, 6)	1 (1, 1)	1 (1, 86)	
Missing	12	25	13	2	2	54	
**Suicidal thoughts**	40 (20.7)	106 (19.0)	70 (26.5)	20 (32.8)	1 (7.1)	237 (21.7)	0.018
**Farewell letter**	17 (8.8)	74 (13.3)	46 (17.4)	18 (29.5)	1 (7.1)	156 (14.3)	0.001
**Trigger**							
Work	11 (5.7)	37 (6.7)	19 (7.4)	4 (6.6)	0 (0)	71 (6.6)	<0.001
Family	28 (14.6)	45 (8.2)	15 (5.8)	13 (21.3)	0 (0)	101 (9.4)	
Financial problems	4 (2.1)	13 (2.4)	9 (3.5)	3 (4.9)	1 (7.1)	30 (2.8)	
Law/Justice	4 (2.1)	10 (1.8)	8 (3.1)	1 (1.6)	0 (0)	23 (2.1)	
Health	13 (6.8)	39 (7.1)	26 (10.1)	3 (4.9)	2 (14.3)	83 (7.7)	
Partner	66 (34.4)	193 (35.0)	70 (27.1)	7 (11.5)	1 (7.1)	338 (31.4)	
Social environment	7 (3.6)	38 (6.9)	10 (3.9)	2 (3.3)	1 (7.1)	337 (31.3)	
Loss of attachment figure/pet animal	3 (1.6)	20 (3.6)	12 (4.7)	1 (1.6)	0 (0)	58 (5.4)	
No trigger	56 (29.2)	157 (28.4)	89 (34.5)	27 (44.3)	9 (64.3)	36 (3.3)	

Unadjusted p-values are shown.

### Relationship between (pre-)clinical parameters and severity of poisoning

Table 3 shows the (pre)clinical parameters in relation to the severity of poisoning (the results of pairwise comparisons are shown in S4 Table). Significant differences were detected in all parameters except for illegal drug co-ingestion. As expected, there was a moderate negative correlation of initial Glasgow Coma Scale (GCS) score with the severity of poisoning (Spearman’s rho -0.437). Furthermore, we observed a moderate positive correlation between the severity of poisoning and duration of inpatient therapy (Spearman’s rho 0.454) and duration of ventilation (Spearman’s rho 0.474), while rescue time (time until admission to hospital) showed only a very weak correlation (Spearman’s rho -0.088). Alcohol was most frequently co-ingested by patients with minor poisoning (35.8%) and least often by those with fatal poisoning (7.1%). The number of classes of substances ingested for suicide attempt correlated weakly with severity (Spearman’s rho 0.109). For absent or mild levels of intoxication (PSS none, minor), an average of 1.75 and 1.78 substances, respectively, were ingested, whereas for higher levels of severity (PSS moderate, severe, fatal), an average of at least two substances were used in suicide attempt. In terms of substance class used in the suicide attempt, antidepressants (most often citalopram and venlafaxin (6 cases, both), trimipramin (4 cases) and amitriptylin, mirtazapin, opipramol, doxepin (3 cases, each)) and cardiovascular drugs (most often antihypertensives including diuretics), predominated within higher severity levels (PSS moderate, severe, fatal, S2 Table). Importantly, 17 patients (25.4%) that used a cardiovascular medication took two or more drugs from this class; 4 of them had a severe or fatal outcome. Furthermore, three fatal cases were documented among the patients that used an insecticide, rodenticide or a metabolic drug (statin and L-thyroxine). Of note, 10 patients (63%) that used a metabolic drug also took a cardiovascular medication (also the only fatal case in connection with metabolic drugs). Anticonvulsants and neuroleptics dominated within moderate levels of poisoning (PSS moderate, severe). Among anticoagulants, the largest proportion was found within severe poisonings (PSS severe). PSS none dominated in the group of patients that used non-opioid analgesics, whereas this severity level was least frequent among the patients that used benzodiazepines.

**Table 3:** Relationship between (pre-)clinical parameters and severity of poisoning.

	None (n = 193)	Minor (n = 558)	Moderate (n = 264)	Severe (n = 61)	Fatal (n = 14)	Total (n = 1090)	p-value
**Initial GCS**							
Mean	14.4	13.6	11.1	8.4	7.9	12.8	<0.001
Median (min, max)	15 (3, 15)	15 (3, 15)	13 (3, 15)	8 (3, 15)	8 (3, 15)	15 (3, 15)	
Missing	40	118	46	22	3	229	
**Rescue time (time until hospital admission)**							
<1 h	16 (11.8)	47 (12.0)	18 (12.2)	2 (8.0)	1 (16.7)	84 (11.9)	0.019
1–3 h	67 (49.3)	179 (45.8)	64 (43.5)	11 (44.0)	1 (16.7)	322 (45.7)	
3–6 h	20 (14.7)	61 (15.6)	16 (10.9)	0 (0)	0 (0)	97 (13.8)	
>6 h	33 (24.3)	104 (26.6)	49 (33.3)	12 (48.0)	4 (66.7)	202 (28.7)	
Missing	57	167	117	36	8	385	
**Co-ingestion of alcohol**	53 (27.5)	200 (35.8)	70 (26.5)	14 (23.0)	1 (7.1)	338 (31.0)	0.004
**Co-ingestion of illegal drugs**	1 (0.5)	9 (1.6)	5 (1.9)	1 (1.6)	0 (0)	16 (1.5)	0.701
**Administration of activated charcoal**	15 (7.8)	35 (6.3)	35 (13.3)	9 (14.8)	3 (21.4)	97 (8.9)	0.002
**Administration of antidote**	19 (9.8)	51 (9.1)	73 (27.7)	27 (44.3)	8 (57.1)	178 (16.3)	<0.001
**Hemodialysis**	0 (0)	3 (0.5)	8 (3.0)	9 (14.8)	4 (28.6)	24 (2.2)	<0.001
**Artificial ventilation**	0 (0)	6 (1.1)	65 (24.6)	41 (67.2)	9 (69.2)	121 (11.1)	<0.001
Missing	0	0	0	0	1	1	
**ICU Treatment**	25 (13.0)	106 (19.0)	158 (59.8)	53 (86.9)	13 (92.9)	355 (32.6)	<0.001
**Duration of artificial ventilation (h)**							
Mean	0.0	0.6	22.0	79.1	68.0	10.9	<0.001
Median (min, max)	0 (0, 0)	0 (0, 144)	0 (0, 336)	45 (0, 500)	5 (0, 504)	0 (0, 504)	
Missing	0	0	0	0	1	1	
**Duration of inpatient treatment**							
≤24 h	99 (51.3)	188 (33.7)	23 (8.7)	2 (3.3)	8 (57.1)	320 (29.4)	<0.001
25–48 h	47 (24.4)	140 (25.1)	35 (13.3)	1 (1.6)	1 (7.1)	224 (20.6)	
49–72 h	21 (10.9)	92 (16.5)	51 (19.3)	3 (4.9)	0 (0)	167 (15.3)	
73–96 h	15 (7.8)	46 (8.2)	33 (12.5)	3 (4.9)	0 (0)	97 (8.9)	
>96 h	11 (5.7)	92 (16.5)	122 (46.2)	52 (85.2)	5 (35.7)	282 (25.9)	
**Number of classes of substances used in the suicide attempt**							
Mean	1.8	1.8	2.1	2.5	2.3	1.9	<0.001
Median (min, max)	1 (1, 7)	1 (1, 8)	2 (1, 13)	2 (1, 8)	1.5 (1, 7)	1 (1, 13)	
Missing	2	2	0	0	0	4	
**Distancing, first psychiatric exploration**	128 (70.3)	315 (57.8)	110 (44.0)	29 (50.9)	0 (0)	582 (56.2)	<0.001
Missing	11	13	14	4	12	54	
**Distancing, second psychiatric exploration**	93 (76.2)	201 (68.6)	101 (62.0)	32 (61.5)	0 (0)	427 (67.7)	0.036
Missing	71	265	101	9	13	459	
**Follow-up therapy**							
Outpatient psychiatric care	10 (5.2)	14 (2.5)	5 (1.9)	2 (3.3)	0 (0)	31	<0.001
Inpatient psychiatric care	87 (45.1)	281 (50.4)	191 (72.3)	49 (80.3)	1 (7.1)	609	
Discharge against medical advice	61 (31.6)	176 (31.5)	42 (15.9)	7 (11.5)	0 (0)	286	
Discharged home	34 (17.6)	80 (14.3)	20 (7.6)	2 (3.3)	0 (0)	136	
Other therapy	1 (0.5)	7 (1.3)	6 (2.3)	1 (1.6)	1 (7.1)	16	
Deceased	0 (0)	0 (0)	0 (0)	0 (0)	12 (85.7)	12	

Data are n (%), unless otherwise indicated. Percentages may not total 100% due to rounding. Unadjusted p-values are shown. GCS, Glasgow Coma Scale; PD, psychiatric disorder. Distancing at first/second psychiatric exploration indicates that patient denied acute suicidality to the consulting psychiatrist at the first or second psychiatric exploration.

In non-fatal cases, patients with PSS none or minor more frequently distanced themselves from the suicide attempt (i.e. denied acute suicidality to the consulting psychiatrist) in both the first and second psychiatric exploration after current suicide attempt than patients with higher levels of severity. Regarding the follow-up treatment, placement in inpatient follow-up treatment was most common within all severity levels and its proportion increased along with the level of severity (Table 3).

### Predictors of a severe or fatal course of suicidal self-poisoning

**Fig 2:**
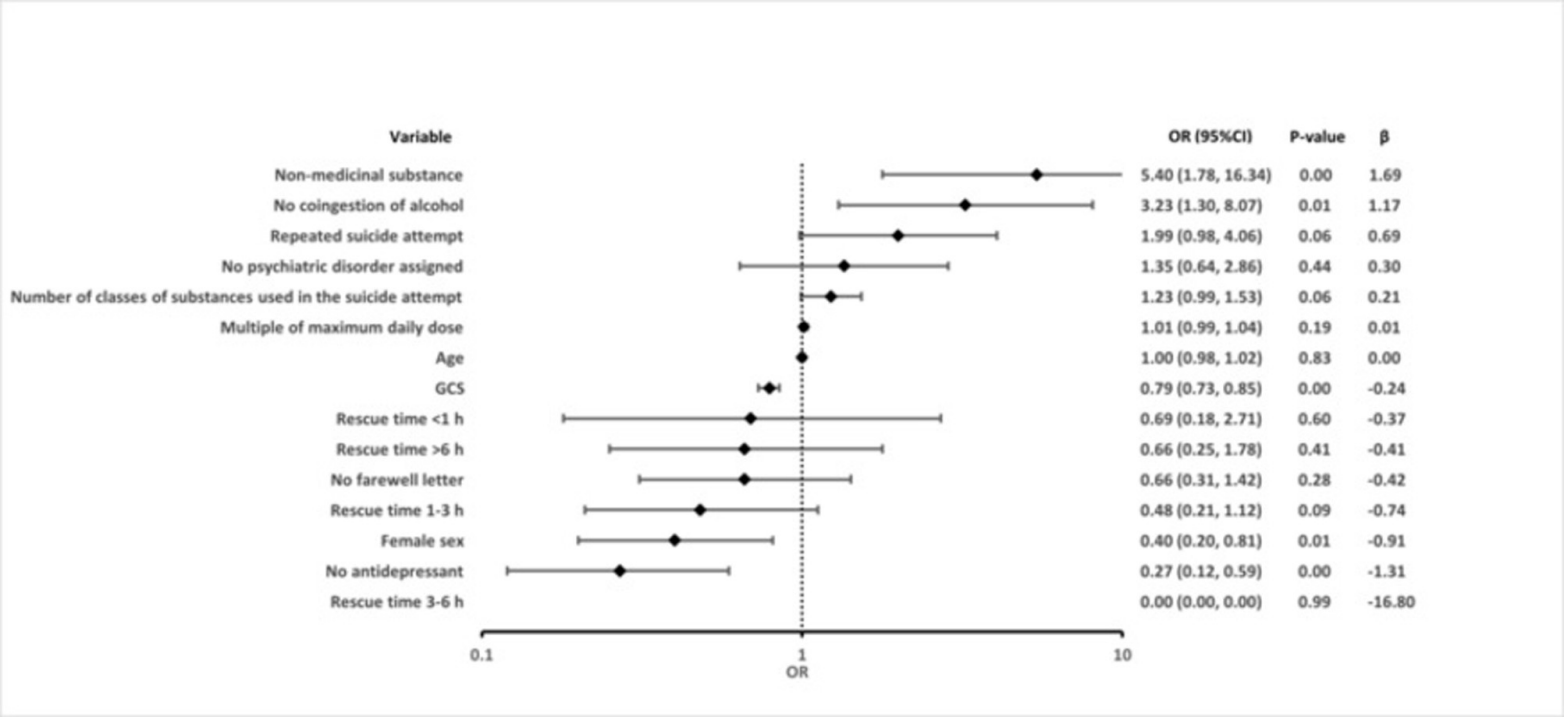
Multiple binomial logistic regression analysis for prediction of severe outcome or death due to self-poisoning. Category “non-medicinal substance” includes car exhaust/carbon monoxide, rodenticide, chemical, plant, insecticide, cleaning agent, drug (ethanol). Multiple of maximum daily dose (MDD) indicates how many times the MDD was crossed for the substance used in self-poisoning. In cases when two or more substances were used, the highest multiple of MDD has been used.

The odds for severe outcome or death were lower by 73% in patients that did not use antidepressants in the suicide attempt (OR 0.27; 95%CI 0.12, 0.59; p = 0.001), by 60% in females (OR 0.4; 95%CI 0.2, 0.81; p = 0.011) and by 21% for each increase of GCS score by one unit (OR 0.79; 95%CI 0.73, 0.85; p<0.001, Fig 2 and S3 Table). Conversely, the risk for severe or fatal outcome was increased more than three-fold when no alcohol was co-ingested (OR 3.23; 95%CI 1.3, 8.07; p = 0.012) or more than five-fold when non-medicinal substances (car exhaust/carbon monoxide, rodenticide, chemical, plant, insecticide, cleaning agent, drug (ethanol)) were used in SRB (OR 5.4; 95%CI 1.78, 16.34; p = 0.003). The remaining factors did not show statistically significant differences of both groups. There were no patients with severe or fatal outcome and rescue time between three to six hours, so no calculation was possible for this variable (rescue time 3-6h).

## Discussion

This large retrospective study aimed to determine factors associated with severe/fatal outcome in patients hospitalized due to deliberate self-poisoning. Our adjusted treatment of patients from mild to life-threatening poisoning in one unit together with timely psychiatric diagnosis and therapy allowed us to determine risk factors in a broad spectrum of patients which are normally treated in different health care facilities according to their severity level. The main findings of our study are: (i) 6.9% of suicide cases had a severe or fatal outcome, (ii) the level of severity increased with age, (iii) severity was similar in patients with and without PD, however, those with mood disorders accounted for more than one third of severe and fatal cases, (iv) severity of poisoning correlated positively with length of inpatient stay and duration of artificial ventilation and negatively correlated with initial GCS, and (v) absence of ethanol co-ingestion and self-poisoning involving antidepressants or non-medicinal substances are predictors of severe/fatal outcome, while female gender and a high initial GCS score were associated with a lower risk of severe/fatal outcome.

Thus far, several models of risk factors for suicide have been described in the literature [17, 18]. However, there is only limited evidence on predictive factors associated specifically with severe or fatal self-poisoning. Co-ingestion of alcohol for intentional self-poisoning has been reported in the literature in about one third of cases [1, 19] and it varies within genders, age groups, and severity levels of poisoning. In our study, co-ingestion of alcohol was least frequent in fatal cases and had the highest proportion within the minor severity group. In contrast, Powell et al., found that both chronic alcoholism and alcohol consumption in the last three hours before the suicide attempt were significantly associated with a nearly lethal suicide attempt [20]. Interestingly, patients included in that study were relatively young (up to 34 years) whereas more than half of our patients were 39 years or older and the severity of suicide weakly correlated with older age. Therefore, it appears this discrepancy in the association between alcohol co-ingestion and suicide severity could be attributed to the differences in the age of patients. Nevertheless, other factors could also play a role since the background of the SRB was underexplored in both studies. For instance, it is unknown whether the co-ingestion of alcohol was planned in the sense of a conscious “courageous drinking”, or the suicide attempt was undertaken as a consequence of an alcohol-related disinhibition. The fact that patients without alcohol co-ingestion are at risk of more severe suicide outcome may reflect their determination to commit suicide. Conversely, patients under the influence of alcohol are more likely to act in an affect-triggered manner while those less determined may “build up their courage” before carrying out their plan. Finally, co-ingestion of alcohol could also reflect a co-existing addictive disorder, which is known to be associated with increased suicidality (see below, [21–23]).

Self-poisoning with non-medicinal substance was also identified as a prognostic factor for more severe suicide outcome in our study. Three out of 14 patients who died due to SRB used an insecticide, rodenticide, or a corrosive agent. Considering insecticides or rodenticides more broadly as agricultural chemicals, the results of this work are comparable to previous studies with elderly patients in which intoxication with agrochemical was a risk factor for poor prognosis, particularly in the Asian population [24–27]. In such cases, there is only a narrow therapeutic window, and a severe toxic effect can be assumed even at low doses. The fact that some patients used non-medicinal substance may indicate untreated or undertreated psychiatric illness. For example, low rates of antidepressant prescribing was reported among Swedish patients who later committed suicide [24]. Furthermore, one-third of fatal cases in our study had no PD which suggests a high rate of underdiagnosis.

In contrast to taking a non-medicinal substance, SRB involving a medication may represent an attempt at self-therapy in the sense of “wanting to rest” or “finally being able to fall asleep” rather than a death wish. We observed that self-poisoning with antidepressants, cardiovascular drugs, anticonvulsants, neuroleptics, anticoagulants and metabolic drugs (e.g. statins and L-thyroxine) was more frequent in moderate and more severe suicide outcome groups. Importantly, self-poisoning with substances other than antidepressants was predictive for better outcome since use of antidepressants was clustered among the fatal courses. Previous studies identified antidepressants, primarily tri- and tetracyclic antidepressants as a risk factor for severe suicide [25, 28, 29]. Pfeifer et al. reported recently that among the patients who attempted a suicide with a psychopharmacological agent, tricyclics had a highest mortality rate among the antidepressants [30]. A recent study investigating suicide attempts by self-poisoning among children and young adults in the United States in 2000–2018 period demonstrated that antidepressants were among the most common substances used in SRB leading to a serious medical outcome [31]. Furthermore, Novack et al. showed that suicide attempt with an antihypertensive drug was a risk factor for ICU stay [32]. A higher severity level associated with these substances could be attributed not only to their toxicity but could also reflect a significant comorbidity since patients in our study frequently used continuous/on demand medication, in line with previous reports [29, 31]. Additionally, the number of classes of substances used in suicide attempt was similar as reported previously [6] and it showed a weak increasing trend with severity level.

Furthermore, a high initial GCS and female gender predicted a less severe or non-fatal self-poisoning. In line with our findings, it was previously shown that a low GCS at clinic presentation was a risk factor for intensive care unit stay following the self-poisoning in Israel [32]. Furthermore, low GCS was associated with poor prognosis of self-poisoning in elderly patients in Republic of Korea [20, 26]. Concerning the relationship between gender and the severity of poisoning it is well known that SRB in males, including self-poisoning, is often characterized by more serious outcome [33–35]. For instance, lethal suicide attempts involving the use of pesticides are more often observed in males than in females, and this effect becomes more pronounced with age [27]. Furthermore, intentional drug overdose leading to more serious outcome or death appears to occur more often in males than females [34, 35].

In addition to these predictive factors identified in the regression analysis, we observed further characteristics associated with severe/fatal outcome. For instance, we noticed that some patients with completed suicide were hospitalized for up to 24 hours while the remaining patients with fatal outcome stayed in the hospital for four days or longer. Thus, death in these patients may be caused by a rapid demise due to a direct toxic effect or it may be caused by complications such as aspiration pneumonia or septicemia. Previous study investigating self-poisoning in England reported the median time to death after hospital admission to be three days with an interquartile range of one to nine days [25]. Furthermore, the proportion of somatic pre-existing conditions was increased within the higher severity levels. This suggests that poisoning can take a severe course mainly caused by coexisting pre-existing conditions. Alternatively, a high comorbidity is an independent risk factor for suicidal behavior. Previously, somatic and several psychiatric disorders were associated with SRB in patients older than 65 years [36]. In our study, mood disorders were diagnosed in more than one-third of patients with moderate, severe, or fatal outcome. The association of mood disorders and severe suicide attempt is well known [21, 36–38]. For instance, Gvion and colleagues identified that major depression was associated with a serious suicide attempt [21]. Interestingly, more than one-third of patients with completed suicide had no diagnosed psychiatric illness. Although this could indicate psychiatric underdiagnosis in these patients, a potential bias due to lack of opportunity for psychiatric evaluation cannot be excluded. The former is supported by the research of Frei et al. who demonstrated that the majority of individuals with completed suicide had no contact with the mental health system [39]. Regarding trigger factors, patients with non-severe or minor outcome attempted suicide due to partner conflicts whereas in those with moderate to fatal outcome, no trigger could have been identified, followed by family and health complaints. Additionally, half or more of patients with PSS moderate, severe and fatal had children. This could be explained by the fact that middle aged patients, who are typically engaged in the parthood, also dominated in these PSS groups. Overall, these findings, along with the higher severity levels within higher age groups and within men corroborate previous observations indicating that women and young people tend to commit a less severe suicide attempt due to interpersonal problems, whereas men and older persons tend to commit a more severe suicide attempt due to personal problems [20, 37, 40].

The strengths of the study include a large sample size, and the fact that the same treatment team and consultant psychiatrists cared for patients throughout the study. Our study has also several limitations. First, data on ingested substances, pre-existing diseases and PD, and details on circumstances of the suicide were incomplete for some patients or they were based on information obtained from third parties. In particular, a potential bias regarding the association of suicide severity and anamnestic or (pre)clinical data cannot be excluded in lethal cases given the lack of opportunity for psychiatric evaluation in these patients. Second, given the low number of severe or fatal cases, the associations detected within our regression analysis should be interpreted with caution. Third, almost half of patients used more than one type of drug which precludes the determination whether a specific substance or their combination led to a severe or fatal outcome. Furthermore, a number of patients used several drugs from the same class, however, they were included only once in the statistical analysis. Since our study has been performed between 2012 and 2016, it may not fully reflect the current clinical practice, especially with regard to the range of substances used. Although the number of completed suicides by self-poisoning in Germany slightly decreased from 2.2–2.4 cases per 100 000 (age standardized) in the 2012–2016 period to 1.8–1.9 cases per 100 000 in the 2017–2020 period [41], to our best knowledge, there is no available information on the frequency and course of all suicide attempts involving self-poisoning (completed plus non-fatal) over the last decade. Finally, since the study did not test a pre-specified hypothesis but rather aimed to identify interactions between several diverse parameters and the severity of suicide, some of the observed effects could be random due to multiple testing.

## Conclusion

Our study demonstrated that male patients hospitalized due to self-poisoning who did not co-ingest alcohol, attempted suicide with non-pharmaceutical substances or antidepressants and had a low initial GCS score are at a higher risk of severe/fatal outcome. Knowledge of these risk factors for a severe or fatal course of poisoning identified in the present study enables the emergency and intensive care physician to better assess the severity and course of poisoning and thus immediately take appropriate measures, such as timely intensive care transfer or transfer to a centre with toxicological expertise and advanced therapeutic options (e.g. extracorporeal toxin elimination and extracorporeal membrane oxygenation).

## Supporting information

S1 Table Definition of PD terms used throughout the study. (TIF)

S2 Table Relationship between substance type used in the suicide attempt and severity of poisoning. (TIF)

S3 Table Multiple binomial logistic regression analysis for prediction of severe outcome or death due to self-poisoning. (TIF)

S4 Table P-values of pairwise comparison between severity of poisoning and different patient characteristics listed in Tables 1–3. (TIF)

S1 Fig Age distribution within the severity levels grouped by gender. (TIF)
