# Supplementary figures and images for: Characteristics and predictive factors of severe or fatal suicide outcome in patients hospitalized due to deliberate self-poisoning
Source: PLoS One. 2022 Nov 3;17(11):e0276000. doi: 10.1371/journal.pone.0276000 (PMC9632874; doi:10.1371/journal.pone.0276000)

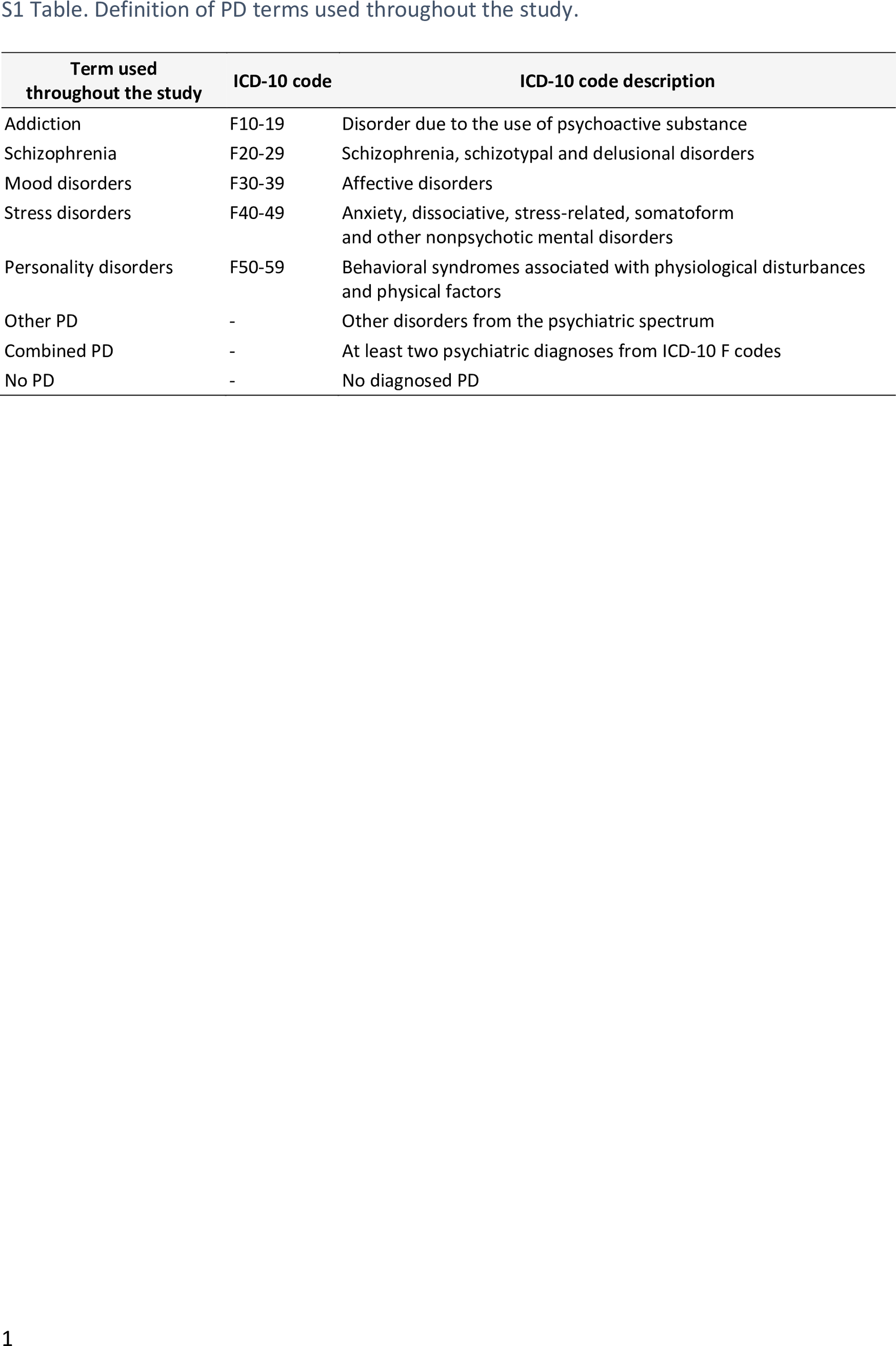

Supplement: S1 Table — (TIF) [file pone.0276000.s001.tif]

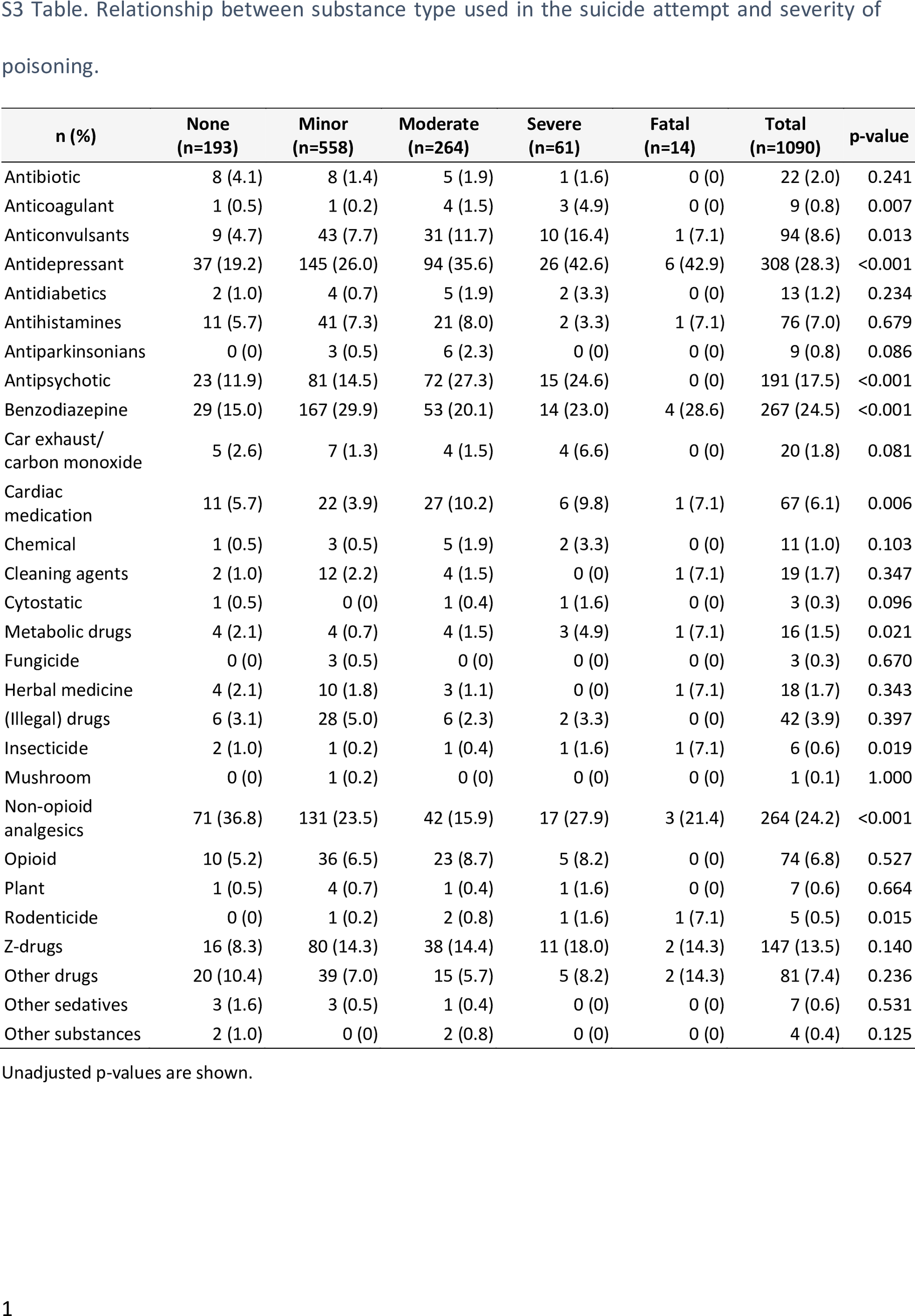

Supplement: S2 Table — (TIF) [file pone.0276000.s002.tif]

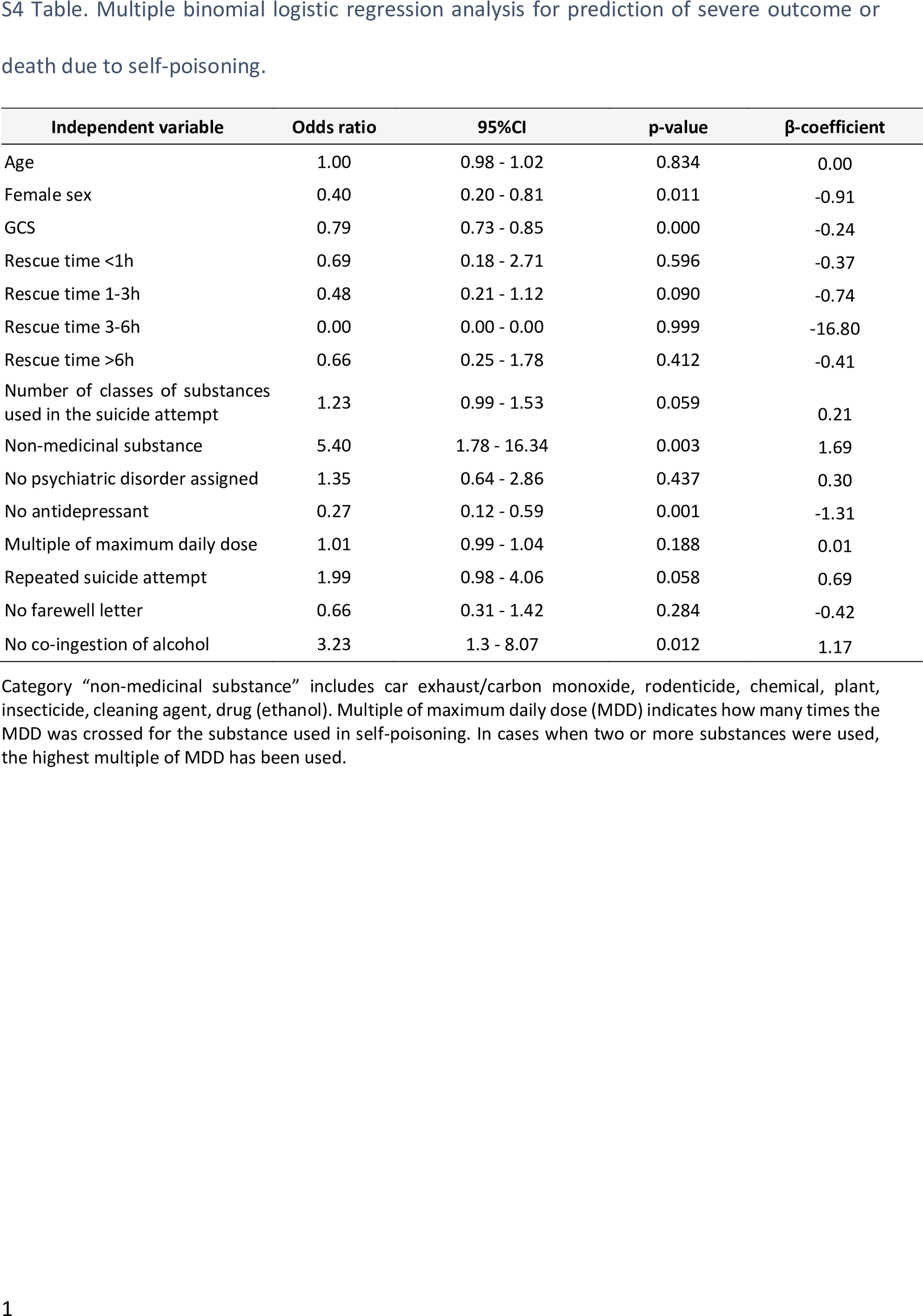

Supplement: S3 Table — (TIF) [file pone.0276000.s003.tif]

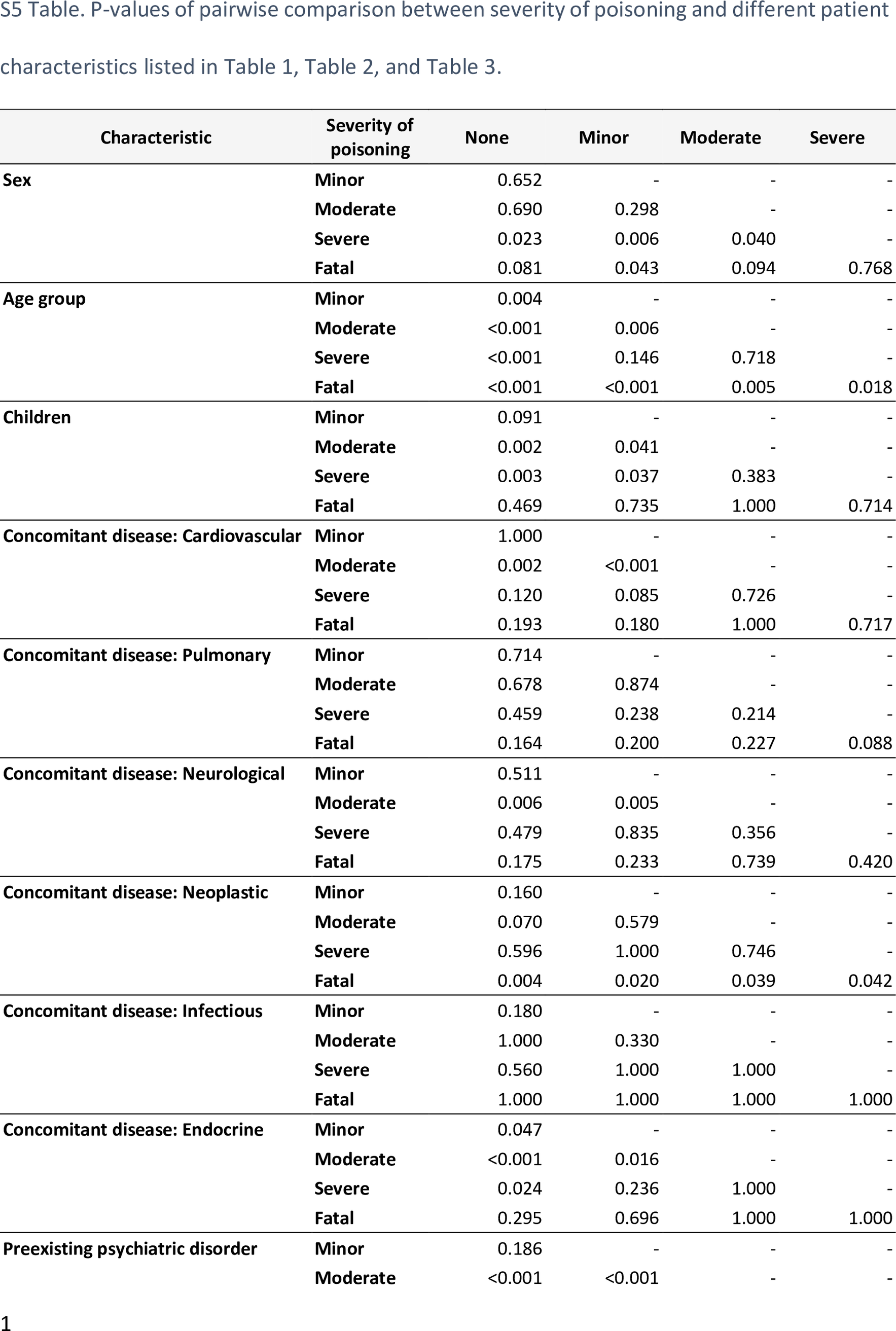

Supplement: S4 Table — (TIF) [file pone.0276000.s004.tif]

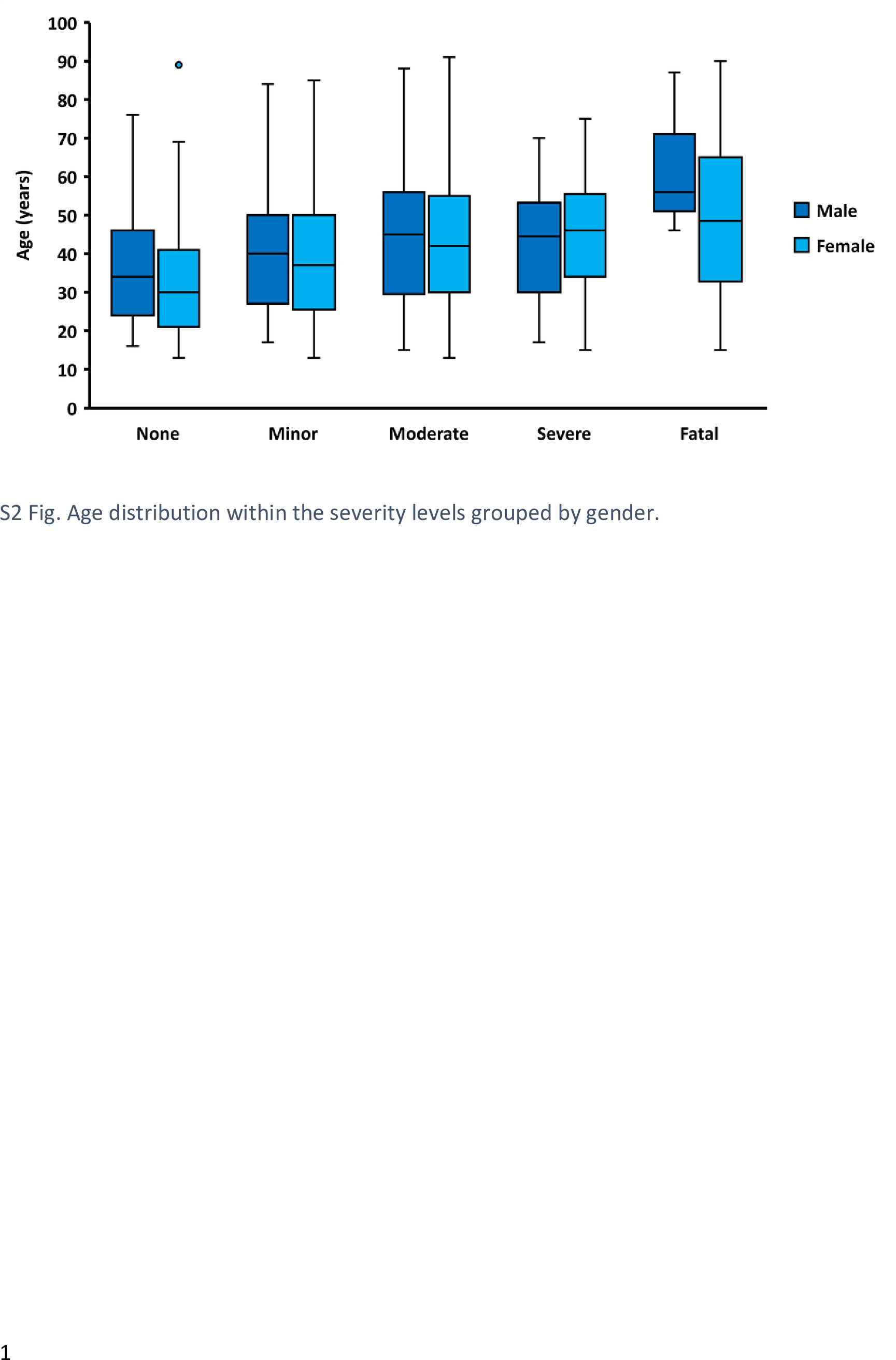

Supplement: S1 Fig — (TIF) [file pone.0276000.s005.tif]
